# Sense of Belonging and Intent to Leave Among Medical School Faculty

**DOI:** 10.1001/jamanetworkopen.2025.7728

**Published:** 2025-04-23

**Authors:** Emily M. Silver, Katherine Balas, Elizabeth H. Ellinas, Jeremy W. Jacobs, Garrett S. Booth, Valerie M. Dandar, Julie K. Silver

**Affiliations:** 1 Department of Psychology, University of Chicago, Chicago, Illinois; 2 Medical School Operations, Association of American Medical Colleges, Washington, DC; 3 Department of Anesthesiology, Medical College of Wisconsin, Milwaukee; 4 Department of Pathology, Microbiology, and Immunology, Vanderbilt University Medical Center, Nashville, Tennessee; 5 Medical School Operations, Association of American Medical Colleges, Washington, DC; 6 Department of Orthopaedic Surgery and Rehabilitation, Wake Forest University School of Medicine, Winston-Salem, North Carolina

## Abstract

This cross-sectional study evaluates sense of belonging and intent to leave their medical school among US medical school faculty.

## Introduction

People seek environments and connections to people, places, and things where they feel a sense of belonging—a basic human need.^1^ Belonging is a feeling whereby an individual believes they are a valued and fundamental part of their surroundings, including family, peer group, occupation, community, or cultural system.^1^

Belonging is believed to be a critical component of workforce retention in medicine, including but not limited to physicians,^2^ and has been associated with intent to leave in various groups.^3,4^ We assessed whether a lower satisfaction with belonging was associated with greater intent to leave their medical school among academic medicine faculty (physicians and other faculty). We also explored rates of satisfaction with belonging by different characteristics (eg, gender, race, and department type) that might impact one's experiences.

## Methods

This cross-sectional study used the Association of American Medical Colleges (AAMC)’s StandPoint Faculty Engagement Survey (SFES), which includes 17 domains reflecting factors known to be associated with faculty engagement and retention. Survey responses from March 2021 to June 2024 were used. Schools contract with the AAMC to administer the survey to faculty. The AAMC’s institutional research board, the American Institutes of Research, approved this project. The first page of this voluntary survey included disclosure language whereby faculty acknowledged their informed consent by continuing to participate. Additional details are provided in the eMethods in Supplement 1 regarding the survey background, analysis, and study population. We followed the Strengthening the Reporting of Observational Studies in Epidemiology (STROBE) reporting guidelines.

Individual-level demographics for responses to the questions: “I am satisfied with my sense of belonging within my department,” and “How likely are you to leave this medical school in the next 1-2 years” were analyzed using 2-tailed *t* tests, analyses of variance, Bonferroni post hoc tests, and odds ratios (ORs) in SPSS version 29 (IBM). A *P* *≤* .05 was significant.

## Results

Survey responses were from 26 US medical schools; the response rate was 62.5% (15 915 of 25 465 faculty). When adjusting for institution, individuals with a neutral satisfaction with belonging were approximately 3 more times likely to report intent to leave (adjusted OR [aOR], 2.84; 95% CI, 2.55-3.15; *P* < .001), and those dissatisfied were 7 times more likely compared with those satisfied with their belonging (aOR, 7.06; 95% CI, 6.28-7.93; *P* < .001) (Table 1). This pattern of increased intent to leave as satisfaction with belonging decreased was consistent across faculty groups. Among those not satisfied, in many cases, the likelihood of leaving was higher for men than women. However, women identified with racial and ethnic minoritized groups and in clinical departments not practicing patient care had higher odds of intent to leave when not satisfied with their belonging compared with men in the same groups. Satisfaction with belonging varied based on factors such as gender, race, and department type (Table 2). Women, as compared with men reported significantly less satisfaction with their sense of belonging (*t*_13 837.02_ = 8.76, *P* < .001); differences within gender existed across races and department types.

**Table 1.** Self-Reported Intention to Leave One’s Medical School by Reported Sense of Belonging Across Gender, Race, and Department Type^a^

No. replied to belonging and intent to leave items	No. (% out of group)		Unadjusted OR (95% CI)	*P* value	aOR (95% CI)	*P* value
	Highly likely, likely, or somewhat likely to leave	Unlikely or highly unlikely to leave				
Total (N = 13 267)						
Satisfied	1905 (19.2)	797 (80.8)	1 [Reference]	NA	1 [Reference]	NA
Neutral	741 (40.0)	111 (60.0)	2.80 (2.52-3.11)	<.001	2.84 (2.55-3.15)	<.001
Dissatisfied	940 (62.1)	573 (37.9)	6.89 (6.14-7.73)	<.001	7.06 (6.28-7.93)	<.001
Gender identity						
Men (n = 7137)						
Satisfied	1009 (18.3)	4495 (81.7)	1 [Reference]	NA	1 [Reference]	NA
Neutral	371 (41.3)	528 (58.7)	3.13 (2.70-3.63)	<.001	3.21 (2.76-3.74)	<.001
Dissatisfied	455 (62.0)	279 (38.0)	7.27 (6.17-8.56)	<.001	7.52 (6.37-8.88)	<.001
Women (n = 6031)						
Satisfied	881 (20.3)	3457 (79.7)	1 [Reference]	NA	1 [Reference]	NA
Neutral	361 (38.8)	570 (61.2)	2.49 (2.14-2.89)	<.001	2.52 (2.16-2.93)	<.001
Dissatisfied	473 (62.1)	289 (37.9)	6.42 (5.45-7.57)	<.001	6.62 (5.60-7.82)	<.001
Race and ethnicity by gender						
American Indian or Alaskan Native men (n = 15)						
Satisfied	7 (70.0)	3 (30.0)	1 [Reference]	NA	1 [Reference]	NA
Neutral	3 (100)	0	n≤10	NA	NA	NA
Dissatisfied	2 (100)	0	n≤10	NA	NA	NA
American Indian or Alaskan Native women (n = 10)						
Satisfied	3 (42.9)	4 (57.1)	1 [Reference]	NA	1 [Reference]	NA
Neutral	0	1 (100)	n≤10	NA	NA	NA
Dissatisfied	2 (100)	0	n≤10	NA	NA	NA
Asian men (n = 1908)						
Satisfied	252 (16.9)	1242 (83.1)	1 [Reference]	NA	1 [Reference]	NA
Neutral	102 (44.2)	129 (55.8)	3.90 (2.91-5.22)	<.001	3.98 (2.95-5.37)	<.001
Dissatisfied	117 (63.9)	66 (36.1)	8.74 (6.28-12.16)	<.001	8.97 (6.40-12.56)	<.001
Asian women (n = 1551)						
Satisfied	254 (22.0)	900 (78.0)	1 [Reference]	NA	1 [Reference]	NA
Neutral	85 (35.4)	155 (64.6)	1.94 (1.44-2.62)	<.001	1.95 (1.43-2.65)	<.001
Dissatisfied	101 (64.3)	56 (35.7)	6.39 (4.48-9.11)	<.001	7.01 (4.84-10.14)	<.001
Black or African American men (n = 177)						
Satisfied	30 (20.1)	119 (79.9)	1 [Reference]	NA	1 [Reference]	NA
Neutral	5 (26.3)	14 (73.7)	1.42 (0.47-4.24)	NS	2.05 (0.55-7.57)	NS
Dissatisfied	5 (55.6)	4 (44.4)	n≤10	NA	NA	NA
Black or African American women (n = 311)						
Satisfied	42 (18.8)	181 (81.2)	1 [Reference]	NA	1 [Reference]	NA
Neutral	23 (50.0)	23 (50.0)	4.31 (2.21-8.41)	<.001	4.63 (2.24-9.58)	<.001
Dissatisfied	27 (64.3)	15 (35.7)	7.76 (3.80-15.86)	<.001	7.74 (3.60-16.65)	<.001
Hispanic, Latino, and/or of Spanish Origin men (n = 400)						
Satisfied	65 (21.7)	235 (78.3)	1 [Reference]	NA	1 [Reference]	NA
Neutral	23 (45.1)	28 (54.9)	2.97 (1.60-5.50)	<.001	2.71 (1.38-5.33)	.004
Dissatisfied	31 (63.3)	18 (36.7)	6.23 (3.28-11.84)	<.001	6.37 (3.17-12.80)	<.001
Hispanic, Latino, and/or of Spanish Origin women (n = 415)						
Satisfied	60 (20.1)	239 (79.9)	1 [Reference]	NA	1 [Reference]	NA
Neutral	21 (33.3)	42 (66.7)	1.99 (1.10-3.61)	.02	2.09 (1.10-3.97)	.02
Dissatisfied	31 (58.5)	22 (41.5)	5.61 (3.03-10.39)	<.001	6.77 (3.43-13.36)	<.001
Native Hawaiian or Other Pacific Islander men (n = 11)						
Satisfied	3 (42.9)	4 (57.1)	1 [Reference]	NA	1 [Reference]	NA
Neutral	0	1 (100)	n≤10	NA	NA	NA
Dissatisfied	1 (33.3)	2 (66.7)	n≤10	NA	NA	NA
Native Hawaiian or Other Pacific Islander women (n = 8)						
Satisfied	0	5 (100)	1 [Reference]	NA	1 [Reference]	NA
Neutral	0	2 (100)	n≤10	NA	NA	NA
Dissatisfied	0	1 (100)	n≤10	NA	NA	NA
White men (n = 4259)						
Satisfied	607 (18.4)	2683 (81.6)	1 [Reference]	NA	1 [Reference]	NA
Neutral	208 (39.2)	323 (60.8)	2.85 (2.34-3.46)	<.001	2.92 (2.40-3.56)	<.001
Dissatisfied	271 (61.9)	167 (38.1)	7.17 (5.80-8.87)	<.001	7.41 (5.97-9.19)	<.001
White women (n = 3372)						
Satisfied	486 (20.0)	1946 (80.0)	1 [Reference]	NA	1 [Reference]	NA
Neutral	204 (40.6)	299 (59.4)	2.73 (2.23-3.35)	<.001	2.79 (2.27-3.43)	<.001
Dissatisfied	266 (60.9)	171 (39.1)	6.23 (5.02-7.73)	<.001	6.44 (5.17-8.03)	<.001
Other race and/or ethnicity men (n = 37)^b^						
Satisfied	2 (7.4)	25 (92.6)	1 [Reference]	NA	1 [Reference]	NA
Neutral	3 (60.0)	2 (40.0)	n≤10	NA	NA	NA
Dissatisfied	4 (80.0)	1 (20.0)	n≤10	NA	NA	NA
Other race and/or ethnicity women (n = 35)^b^						
Satisfied	3 (18.8)	13 (81.3)	1 [Reference]	NA	1 [Reference]	NA
Neutral	3 (33.3)	6 (66.7)	n≤10	NA	NA	NA
Dissatisfied	7 (70.0)	3 (30.0)	n≤10	NA	NA	NA
≥2 Races and/or ethnicities men (n = 214)						
Satisfied	28 (17.7)	130 (82.3)	1 [Reference]	NA	1 [Reference]	NA
Neutral	13 (43.3)	17 (56.7)	3.55 (1.55-8.14)	.003	4.56 (1.74-11.99)	.002
Dissatisfied	12 (46.2)	14 (53.8)	3.98 (1.66-9.52)	.002	4.18 (1.61-10.87)	.003
≥2 Races and/or ethnicities women (n = 220)						
Satisfied	23 (15.2)	128 (84.8)	1 [Reference]	NA	1 [Reference]	NA
Neutral	16 (51.6)	15 (48.4)	5.94 (2.58-13.65)	<.001	6.38 (2.52-16.15)	<.001
Dissatisfied	21 (55.3)	17 (44.7)	6.88 (3.16-14.97)	<.001	6.34 (2.68-15.02)	<.001
Department type and clinical activity by gender						
Basic science men (n = 1146)						
Satisfied	149 (17.3)	710 (82.7)	1 [Reference]	NA	1 [Reference]	NA
Neutral	57 (36.5)	99 (63.5)	2.74 (1.89-3.98)	<.001	2.68 (1.83-3.92)	<.001
Dissatisfied	82 (62.6)	49 (37.4)	7.97 (5.37-11.84)	<.001	8.40 (5.55-12.72)	<.001
Basic science women (n = 760)						
Satisfied	112 (20.6)	431 (79.4)	1 [Reference]	NA	1 [Reference]	NA
Neutral	36 (29.5)	86 (70.5)	1.61 (1.04-2.50)	.03	1.65 (1.04-2.62)	.04
Dissatisfied	53 (55.8)	42 (44.2)	4.86 (3.08-7.66)	<.001	5.07 (3.13-8.23)	<.001
Clinical men active in clinical care (n = 5155)						
Satisfied	755 (18.7)	3284 (81.3)	1 [Reference]	NA	1 [Reference]	NA
Neutral	258 (43.0)	342 (57.0)	3.28 (2.74-3.93)	<.001	3.42 (2.85-4.11)	<.001
Dissatisfied	328 (63.6)	188 (36.4)	7.59 (6.24-9.23)	<.001	8.00 (6.55-9.77)	<.001
Clinical women active in clinical care (n = 4627)						
Satisfied	703 (20.8)	2680 (79.2)	1 [Reference]	NA	1 [Reference]	NA
Neutral	286 (41.9)	396 (58.1)	2.75 (2.32-3.27)	<.001	2.83 (2.37-3.37)	<.001
Dissatisfied	358 (63.7)	204 (36.3)	6.69 (5.53-8.10)	<.001	6.89 (5.67-8.37)	<.001
Clinical men not active in clinical care (n = 821)						
Satisfied	103 (17.4)	490 (82.6)	1 [Reference]	NA	1 [Reference]	NA
Neutral	55 (39.0)	86 (61.0)	3.04 (2.04-4.54)	<.001	3.02 (1.99-4.58)	<.001
Dissatisfied	45 (51.7)	42 (48.3)	5.10 (3.18-8.17)	<.001	5.47 (3.34-8.97)	<.001
Clinical women not active in clinical care (n = 630)						
Satisfied	64 (15.9)	339 (84.1)	1 [Reference]	NA	1 [Reference]	NA
Neutral	37 (30.1)	86 (69.9)	2.28 (1.43-3.64)	<.001	2.43 (1.49-3.95)	<.001
Dissatisfied	61 (58.7)	43 (41.3)	7.51 (4.68-12.06)	<.001	8.24 (4.96-13.70)	<.001

Abbreviations: aOR, adjusted odds ratio; NA, not applicable; NS, not significant.

^a^ Satisfaction with one’s sense of belonging is defined by response to the survey item “I am satisfied with my sense of belonging within the department,” where satisfied includes those who responded strongly agree or agree, neutral includes those who responded neither agree nor disagree, and dissatisfied includes those who responded disagree or strongly disagree. Testing compared each group (those dissatisfied and separately, those neutral) with those who were satisfied (the reference group within each characteristic group (eg, women dissatisfied with their sense of belonging compared with women satisfied with their sense of belonging). Intention to leave is defined by response to the survey item, “How likely are you to leave this medical school in the next 1-2 years?” where likely to leave indicates those who responded highly likely, likely, or somewhat likely and unlikely to leave indicates those who responded unlikely or not at all likely. Intention to leave does not include those who plan to retire within the next 1 to 2 years. Odds ratios (ORs) used multivariable analyses to adjust for respondent’s medical school in consideration for potential differences and/or variation between schools. ORs and *P* values are not reported where the sample size was equal to or less than 10 individuals.

^b^ Participants selected "other race and ethnicity" on the survey; no additional details are available.

**Table 2.** Distribution of Self-Reported Satisfaction With Sense of Belonging (Per Survey Item “I Am Satisfied With My Sense of Belonging Within My Department”) by Gender, Race, and Department Type^a^

Characteristic^b^	Survey item respondent No.	I am satisfied with my sense of belonging in my department, No. (%)			Descriptive Statistic		
		Strongly agree/agree	Neither agree nor disagree	Strongly disagree/disagree	Mean (SD) [95% CI]	Post hoc comparisons where *P* ≤ .05^b^	*t*-Test/ ANOVA (*P* value)
Total respondents	14 594	10762 (73.7)	2102 (14.4)	1730 (11.9)	3.88 (1.03) [3.86-3.90]	NA	NA
Gender identity							
Men	7934	6053 (76.3)	1033 (13.0)	848 (10.7)	3.95 (1.02) [3.93-3.97]	NA	8.76 (<.001)
Women	6541	4636 (70.9)	1040 (15.9)	865 (13.2)	3.80 (1.04) [3.77-3.82]		
Race and ethnicity							
American Indian or Alaskan Native (a)	30	19 (63.3)	7 (23.3)	4 (13.3)	3.70 (0.95) [3.34-4.06]	NS	3.71 (<.001)
Asian (b)	3687	2791 (75.7)	516 (14.0)	380 (10.3)	3.88 (0.97) [3.85-3.91]	g	
Black or African American (c)	513	387 (75.4)	68 (13.3)	58 (11.3)	3.88 (1.01) [3.80-3.97]	g	
Hispanic, Latino, and/or of Spanish origin (d)	879	634 (72.1)	128 (14.6)	117 (13.3)	3.81 (1.04) [3.75-3.88]	NS	
Native Hawaiian or Other Pacific Islander (e)	21	13 (61.9)	4 (19.0)	4 (19.0)	3.62 (1.02) [3.15-4.08]	NS	
White (f)	8544	6330 (74.1)	1194 (14.0)	1020 (11.9)	3.91 (1.05) [3.89-3.93]	g	
Other race and/or ethnicity (g)^c^	88	54 (61.4)	18 (20.5)	16 (18.2)	3.51 (1.15) [3.27-3.76]	b,c,f	
≥2 Races and/or ethnicities (h)	469	328 (69.9)	70 (14.9)	71 (15.1)	3.80 (1.11) [3.70-3.90]	NS	
Race and ethnicity by gender							
American Indian or Alaskan Native men(a)	16	11 (68.8)	3 (18.8)	2 (12.5)	3.81 (0.98) [3.29-4.34]	NS	7.02 (<.001)
American Indian or Alaskan Native women(b)	13	8 (61.5)	3 (23.1)	2 (15.4)	3.62 (0.96) [3.03-4.20]	NS	
Asian men (c)	2035	1582 (77.7)	249 (12.2)	204 (10.0)	3.92 (0.97) [3.88-3.97]	l,n	
Asian women (d)	1647	1206 (73.2)	266 (16.2)	175 (10.6)	3.83 (0.97) [3.78-3.87]	k	
Black or African American men (e)	180	152 (84.4)	19 (10.6)	9 (5.0)	4.08 (0.83) [3.96-4.20]	n	
Black or African American women (f)	332	235 (70.8)	48 (14.5)	49 (14.8)	3.78 (1.08) [3.66-3.90]	NS	
Hispanic, Latino, and/or of Spanish origin men (g)	440	323 (73.4)	59 (13.4)	58 (13.2)	3.84 (1.08) [3.74-3.94]	NS	
Hispanic, Latino, and/or of Spanish origin women (h)	437	309 (70.7)	69 (15.8)	59 (13.5)	3.79 (1.01) [3.69-3.88]	k	
Native Hawaiian or Other Pacific Islander men (i)	12	8 (66.7)	1 (8.3)	3 (25.0)	3.58 (1.08) [2.89-4.27]	NS	
Native Hawaiian or Other Pacific Islander women (j)	8	5 (62.5)	2 (25.0)	1 (12.5)	3.75 (1.04) [2.88-4.62]	NS	
White men (k)	4839	3693 (76.3)	631 (13.0)	515 (10.6)	3.98 (1.03) [3.95-4.01]	d,h,l,n,p	
White women (l)	3690	2626 (71.2)	561 (15.2)	503 (13.6)	3.81 (1.06) [3.78-3.85]	c,k	
Other race and/or ethnicity men (m)^c^	40	29 (72.5)	6 (15.0)	5 (12.5)	3.68 (1.12) [3.32-4.03]	NS	
Other race and/or ethnicity women (n)^c^	38	18 (47.4)	10 (26.3)	10 (26.3)	3.32 (1.21) [2.92-3.71]	c,e,k	
≥2 Races and/or ethnicities men (o)	229	168 (73.4)	33 (14.4)	28 (12.2)	3.91 (1.02) [3.78-4.04]	NS	
≥2 Races and/or ethnicities women (p)	233	158 (67.8)	35 (15.0)	40 (17.2)	3.73 (1.16) [3.58-3.88]	k	
Department type and clinical activity							
Basic science (a)	2120	1541 (72.7)	315 (14.9)	264 (12.5)	3.86 (1.07) [3.82-3.91]	c	15.19 (<.001)
Clinical-active in clinical care (b)	10645	7980 (75.0)	1433 (13.5)	1232 (11.6)	3.90 (1.03) [3.88-3.92]	c	
Clinical-not active in clinical care (c)	1691	1148 (67.9)	322 (19.0)	221 (13.1)	3.75 (1.04) [3.70-3.80]	a,b	
Department type and clinical activity by gender							
Basic science men (a)	1277	942 (73.8)	179 (14.0)	156 (12.2)	3.89 (1.06) [3.83-3.95]	c,f	23.84 (<.001)
Basic science women (b)	824	584 (70.9)	135 (16.4)	105 (12.7)	3.83 (1.07) [3.75-3.90]	c,f	
Clinical men-active in clinical care (c)	5631	4377 (77.7)	674 (12.0)	580 (10.3)	3.98 (1.01) [3.96-4.01]	a,b,d,e,f	
Clinical women-active in clinical care (d)	4933	3556 (72.1)	739 (15.0)	638 (12.9)	3.82 (1.03) [3.79-3.84]	c,f	
Clinical men-not active in clinical care (e)	970	694 (71.5)	171 (17.6)	105 (10.8)	3.84 (1.00) [3.78-3.91]	c,f	
Clinical women-not active in clinical care (f)	710	447 (63.0)	147 (20.7)	116 (16.3)	3.63 (1.09) [3.55-3.71]	a,b,c,d,e	
Intent to leave medical school							
Highly likely, likely, or somewhat likely	3586	1905 (53.1)	741 (20.7)	940 (26.2)	3.33 (1.18) [3.29-3.37]	NA	36.15 (<.001)
Unlikely or not at all likely	9681	7997 (82.6)	1111 (11.5)	573 (5.9)	4.11 (0.87) [4.09-4.13]		
Intent to leave medical school by gender							
Highly likely, likely, or somewhat likely men (a)	1835	1009 (55.0)	371 (20.2)	455 (24.8)	3.38 (1.18) [3.32-3.43]	b,c,d	590.68 (<.001)
Highly likely, likely, or somewhat likely women (b)	1715	881 (51.4)	361 (21.0)	473 (27.6)	3.29 (1.17) [3.23-3.34]	a,c,d	
Unlikely or not at all likely men (c)	5302	4495 (84.8)	528 (10.0)	279 (5.3)	4.17 (0.85) [4.15-4.20]	a,b,d	
Unlikely or not at all likely women (d)	4316	3457 (80.1)	570 (13.2)	289 (6.7)	4.03 (0.88) [4.00-4.06]	a,b,c	

Abbreviations: ANOVA, analysis of variance; NA, not applicable; NS, not significant.

^a^ Table shows descriptive statistics by select demographics and retention factors for the survey item “I am satisfied with my sense of belonging within my department,” where agreement was self-reported on a 1 through 5 Likert scale with higher scores being more favorable and indicating higher rates of agreement. Intent to leave is assessed based on the survey question, “How likely or unlikely are you to leave this medical school in the next 1-2 years?” which uses a 5-point scale of highly likely to not at all likely, combined here for analysis.

^b^ Notations of a through g are used as indicators within each demographic to identify the comparison groups included within the pairwise post hoc tests. Groups with significant differences are noted in the post hoc comparisons column.

^c^ Participants selected "other race and ethnicity" on the survey; no additional details are available.

## Discussion

This cross-sectional study found that lower satisfaction with belonging is associated with greater intent to leave among faculty, consistent with prior studies.^3,4^ Findings showed that differences in satisfaction with belonging vary by factors such as gender, race, and department type (Table 2). Compared with men, women reported significantly less satisfaction with belonging. Women identified with minoritized racial and ethnic groups and in clinical departments not practicing patient care had higher odds of intent to leave when not satisfied with their belonging compared with men in the same groups.

Our results contribute to literature linking intent to leave with physician well-being.^5^ They also provide additional information about career dissatisfaction that is particularly notable for women faculty.^6^ There is a paucity of studies focused on belonging interventions related to faculty retention.

This study is limited to faculty that completed the SFES and assessment of belonging at the department level. Future work should include other cohorts and measures of belonging.

Faculty retention is essential to a sustainable medical workforce. Identifying cohorts at risk of leaving their institutions provides an opportunity to proactively foster belongingness to retain faculty and optimize medical school recruitment investments. This, in turn, enhances high-quality medical education and patient care as well as the advancement of scientific discovery. Medical schools should design and implement programs that enhance belonging.

## Notes

Supplement 1.
eMethods.
**eTable 1. **StandPoint Survey Schools Compared With All US LCME-Accredited Medical Schools **eTable 2. **StandPoint Survey Respondent Characteristics Compared With All US Full-Time Faculty

Supplement 2.
Data Sharing Statement
