# Supplementary material for: Sense of Belonging and Intent to Leave Among Medical School Faculty
Source: JAMA Netw Open. 2025 Apr 23;8(4):e257728. doi: 10.1001/jamanetworkopen.2025.7728 (PMC12019519; doi:10.1001/jamanetworkopen.2025.7728)
Supplement: Supplement 1. — eMethods. eTable 1. StandPoint Survey Schools Compared With All US LCME-Accredited Medical Schools eTable 2. StandPoint Survey Respondent Characteristics Compared With All US Full-Time Faculty [file jamanetwopen-e257728-s001.pdf]

Supplemental Online Content

Silver EM, Balas K, Ellinas E, et al. Sense of belonging and intent to leave among medical school faculty. *JAMA Netw Open*. 2025;8(4):e257728. doi:10.1001/jamanetworkopen.2025.7728

eMethods

eTable 1. StandPoint Survey Schools Compared With All US LCME-Accredited Medical Schools

eTable 2. StandPoint Survey Respondent Characteristics Compared With All US Full-Time Faculty

This supplemental material has been provided by the authors to give readers additional information about their work.

© 2025 Silver EM et al. *JAMA Network Open*.

eMethods

AAMC StandPoint Faculty Engagement Survey Background

Data used in this analysis come from the Association of American Medical Colleges' (AAMC) StandPoint Faculty Engagement Survey (SFES). The primary purpose of the survey is to help institutions in assessing their workplace, making improvements based on faculty feedback, and ultimately improving faculty engagement and retention. StandPoint Surveys is an optional program offered by the AAMC whereby a school contracts with the AAMC to administer the survey on their behalf. To date, over half of US LCME-accredited medical schools have administered the survey at least once, with some schools regularly administering every three to five years.

The survey was developed in 2008 by experts in academic medicine, survey research, and organizational management and based on focus group research with medical school faculty. First launched in 2009, the survey contains 47 questions, the majority of which use 5-point Likert scales of agreement and satisfaction. Questions are organized into 17 domains, or topic areas, that reflect the factors known to drive faculty engagement and retention, such as medical school governance, relationship with one's supervisor, focus on school mission etc. Additionally, the survey collects various datapoints about a faculty member's professional and personal identities including appointment type, academic rank, department/specialty, degree type, administrative role, gender identity, race/ethnicity, sexual orientation, and age. The survey has been validated numerous times since 2009 to ensure reliability, with the last validation effort taking place in 2020.

The AAMC administers the survey via the web to all full and part-time faculty over a 6-week period of the medical school's choosing. The AAMC creates aggregated reports for the medical school with cohort level benchmarking including data collected over the past three-year period. For schools that might have administered multiple times over the three-year period, only their most recent survey administration is included. The AAMC manages a longitudinal database of StandPoint Survey data that is used for de-identified research, such as this article.

Belonging and Retention Analysis

This analysis uses data from the March 2021-June 2024 administrations of the SFES. Responses to the following two questions were analyzed using a 5-point agreement scale (e.g. Strongly Agree through Strongly Disagree) and 5-point likelihood scale (e.g. Highly Likely through Not at all Likely) respectively: "I am satisfied with my sense of belonging within my department" and "How likely are you to leave this medical school in the next 1-2 years". Data was compared by individual-level demographics including gender identity, race/ethnicity, and department type.

Gender identity is presented in the results as comparing only men and women. While the SFES now collects data about individuals with other gender identities, it did not until 2023. Since the data were not available for the whole study period, those respondents with other gender identities were excluded. There is a single item that allows individuals to indicate the races/ethnicities with which they identify, and individuals can select as many responses as applies. Those data are then aggregated to identify those who selected one or more races/ethnicities. With regards to department, each faculty member is classified as in a basic science or clinical department. Data for faculty in clinical departments is disaggregated based on whether or not they indicated that they actively participate in delivering patient care.

This analysis used descriptive and inferential statistics including reports of frequencies (collapsed to a 3-point scale for reporting), means, t-tests, ANOVAs with Bonferroni Post-Hoc tests, and odds ratios (5-point scale). Each question in the SFES is optional and thus respondents may skip any questions they choose. Response rates vary for each question, with 92% of survey respondents having provided data about their sense of belonging. Intent to leave questions are asked with branching logic based on responses. First, respondents are asked how likely they are to retire in the next 1-2 years. Those who indicate that they are "Highly Likely" or "Likely" to retire are not asked the subsequent question about how likely they are to leave the medical school in the next 1-2 years. Thus, response rates for this question are lower to account for removing those planning to retire. Missing data was excluded from the analysis and no data were imputed.

© 2025 Silver EM et al. *JAMA Network Open*.

Sample Representation

While not all medical schools participate in the program, the AAMC's 2021-2024 cohort of 26 institutions is diverse in terms school ownership type (e.g. public or private), region, organizational structure with affiliated institutions (e.g. hospitals and practice plans), faculty size, mission focus (e.g. research intensive or community-based), as well includes as schools that are newly accredited. Based on the AAMC's Organizational Characteristics Database which contains institutional characteristics data for each medical school, the StandPoint Survey cohort has less institutions located in the Northeast region and more in the Southern region than the distribution by region across all US medical schools. Additionally, the StandPoint Survey cohort has more public schools and community-based schools, and less research-intensive schools.

eTable 1. StandPoint Survey Schools Compared With All US LCME-Accredited Medical Schools

Medical School Characteristics	2021-2024 StandPoint Survey School Cohort (n=26)	All US LCME-Accredited Medical Schools (n=158)
Region		
Central	26.9%	22.2%
Northeast	7.7%	26.6%
Southern	50.0%	36.1%
Western	15.4%	15.2%
Ownership		
Private	26.9%	38.6%
Public	73.1%	61.4%
Additional Characteristics		
Community-Based Medical Schools	30.7%	16.5%
Research-Intensity Rank based on Federal Directs	84	75

Source: AAMC Organizational Characteristics Database, June 2024

The AAMC's Faculty Roster collects data on all full-time faculty across US LCME-accredited medical schools. While the SFES includes both full and part-time faculty, comparing the StandPoint Survey respondent pool with the Faculty Roster provides insight into representation. Compared with the Faculty Roster, the StandPoint cohort has a similar distribution by gender, but slightly more basic science faculty respondents. While there appear to be some differences based on the distribution by race and ethnicity, StandPoint does not include individuals who identify as both Hispanic and White in the multiple races/ethnicities category, whereas the Faculty Roster does include this group in calculations. Thus, it is regularly observed that there are differences in the numbers of Hispanic, Latino, Spanish Origin faculty and faculty of multiple races/ethnicities in comparing the two databases.

eTable 2. StandPoint Survey Respondent Characteristics Compared With All US Full-Time Faculty

Survey Respondent Characteristics	2021-2024 StandPoint Survey School Cohort (n=15,915)	All US LCME-Accredited Medical Schools (n=201,112)
Gender Identity	(15, 737)	(n=200,990)
Men	54.0%	55.5%
Women	46.0%	44.5%
Race/Ethnicity	(n=15,181)	(n=193,308)

© 2025 Silver EM et al. *JAMA Network Open*.

American Indian/Alaskan Native	0.2%	0.2%
Asian	26.2%	22.4%
Black or African American	3.7%	4.1%
Hispanic, Latino, of Spanish Origin	6.2%	3.8%
Native Hawaiian/Other Pacific Islander	0.1%	0.1%
White	59.8%	63.4%
Other Race/Ethnicity	0.6%	1.1%
Multiple Races/Ethnicities	3.2%	5.0%
Department Type	(n=15,915)	(n=197,208)
Basic Science	14.1%	10.0%
Clinical	85.9%	90.0%

Source: AAMC Faculty Roster; December 31, 2023 snapshot as of December 31, 2023.

© 2025 Silver EM et al. *JAMA Network Open*.
