# Supplementary material for: Sense of Belonging and Intent to Leave Among Medical School Faculty
Source: JAMA Netw Open. 2025 Apr 23;8(4):e257728. doi: 10.1001/jamanetworkopen.2025.7728 (PMC12019519; doi:10.1001/jamanetworkopen.2025.7728)
Supplement: Supplement 2. — Data Sharing Statement [file jamanetwopen-e257728-s002.pdf]

Data Sharing Statement

Silver. Sense of Belonging and Intent to Leave Among Medical School Faculty. *JAMA Netw Open*. Published April 23, 2025. doi:10.1001/jamanetworkopen.2025.7728

Data
Data available: Yes
Data types: Deidentified participant data
How to access data: Researchers may request data from AAMC.
When available: With publication

Supporting Documents
Document types: None

Additional Information
Who can access the data: Researchers may request data from AAMC.
Types of analyses: Research
Mechanisms of data availability: After approval from AAMC
